# Combinatorial suicide gene strategies for the safety of cell therapies

**DOI:** 10.3389/fimmu.2022.975233

**Published:** 2022-09-14

**Authors:** Corey Falcon, Lauren Smith, Mustafa Al-Obaidi, Mohammed Abu Zaanona, Katelyn Purvis, Kentaro Minagawa, Mohammad Athar, Donna Salzman, Ravi Bhatia, Frederick Goldman, Antonio Di Stasi

**Affiliations:** Hematology/Oncology, The University of Alabama at Birmingham, Birmingham, AL, United States

**Keywords:** inducible caspase 8, inducible caspase 9, suicide gene, safety switch, RQR8

## Abstract

Gene-modified cellular therapies carry inherent risks of severe and potentially fatal adverse events, including the expansion of alloreactive cells or malignant transformation due to insertional mutagenesis. Strategies to mitigate uncontrolled proliferation of gene-modified cells include co-transfection of a suicide gene, such as the inducible caspase 9 safety switch (ΔiC9). However, the activation of the ΔiC9 fails to completely eliminate all gene-modified cells. Therefore, we tested a two suicide gene system used independently or together, with the goal of complete cell elimination. The first approach combined the ΔiC9 with an inducible caspase 8, ΔiC8, which lacks the endogenous prodomain. The rationale was to use a second caspase with an alternative and complementary mechanism of action. Jurkat cells co-transduced to co-express the ΔiC8, activatable by a BB homodimerizer, and the ΔiC9 activatable by the rapamycin analog sirolimus were used in a model to estimate the degree of inducible cell elimination. We found that both agents could activate each caspase independently, with enhanced elimination with superior reduction in cell regrowth of gene-modified cells when both systems were activated simultaneously. A second approach was employed in parallel, combining the ΔiC9 with the RQR8 compact suicide gene. RQR8 incorporates a CD20 mimotope, targeted by the anti-CD20 monoclonal antibody rituxan, and the QBend10, a ΔCD34 selectable marker. Likewise, enhanced cell elimination with superior reduction in cell regrowth was observed when both systems were activated together. A dose-titration effect was also noted utilizing the BB homodimerizer, whereas sirolimus remained very potent at minimal concentrations. Further *in vivo* studies are needed to validate these novel combination systems, which may play a role in future cancer therapies or regenerative medicine.

## Introduction

Given the recent surge of novel cellular therapies, there is an urgent need to develop strategies to mitigate untoward events of gene-modified cells. Chimeric antigen receptor (CAR) redirected T-cells, gene-modified hematopoietic stem cells (HSC), and inducible pluripotent stem cells (iPSC) are emerging treatments for a wide variety of malignant and non-malignant disorders (1–3). However, gene modifying therapies carry the intrinsic risks of excess proliferation (4, 5), and insertional mutagenesis (6–10). In the case of CAR-T specifically, off-target side effects could result in potentially fatal organ damage and death including immune effector cell-associated neurotoxicity syndrome (ICANS) and severe cytokine release syndrome (CRS) (11). Currently available treatments for CRS are limited to corticosteroids or anti-interleukin-6 receptor antibodies, which are associated with broad immunosuppression.

It is very challenging to predict the type or degree of the toxicities that may occur. For example, injection of even unmodified autologous HSC into the kidneys to treat renal failure resulted in angiomyeloproliferative lesions that required nephrectomy (12). Autologous stem cells derived from adipose tissue and injected intravitreally for macular degeneration were associated with worsening vision in three people, two of whom became legally blind (13). Gene-modified HSC infused into patients with monogenic disease (6, 7, 9, 10, 14), resulted in leukemia from insertional mutagenesis in several patients. This risk is possible also with the use of iPSC, *e.g.*, a patient developed glioneuronal multifocal brain cancer after the infusion of fetal donor-derived neuronal stem cells (4). These detrimental effects could be potentially alleviated by employing a cellular suicide gene strategy in which a gene is inserted into the therapeutic cell and can then be activated ‘on demand’, causing cell death. Suicide gene technologies can be broadly classified based upon their mechanism of action in (i) metabolic (gene-directed enzyme prodrug therapy, (ii) dimerization-induced apoptosis, and (iii) monoclonal antibody-mediated cytotoxicity. However, currently available suicide gene systems are not ideal.

Gene-directed enzyme prodrug therapy converts a nontoxic drug to a toxic drug in gene-modified cells, as with the human herpes-simplex-virus-thymidine-kinase (HSV-TK)/ganciclovir, which is the first invented suicide gene system. The HSV-TK system showed promise but has major limitations. The HSV-TK transgene used as the suicide gene is immunogenic, and its activation requires the administration of the therapeutic anti-viral agent ganciclovir.

Its immunogenicity could preclude persistence and activity of the infused therapeutic cells. In the setting of donor lymphocyte infusion post allogeneic HSCT this still resulted in anti-tumor effect, likely due to the slow kinetic of cell’s elimination (15).

The requirement for ganciclovir may limit its use in patients with cytomegalovirus infection where this agent is a primary treatment option. In addition, ganciclovir has other off-target toxicities, including myelosuppression and renal dysfunction.

The ΔiC9 suicide gene is expectedly less immunogenic, and the activating agent is not a therapeutic drug. Triggering of the ΔiC9 suicide gene leads to the activation of multiple executioner caspases (caspases 3, 6, and 7) and is effective in inducing apoptotic cell death.

The ΔiC9 suicide gene is a chimeric protein composed of a drug-binding domain linked in frame with a component of the apoptotic pathway, allowing conditional dimerization and apoptosis of the transduced cells after administration of a non-therapeutic small molecule dimerizer, such as AP1903 or BB homodimerizer (15–19). Straathof et al. (19) and Tey et al. (20) validated the ΔiC9 construct for T cell applications, demonstrating optimal transduction efficiency, expansion, and elimination of ΔiC9 T cells with strong expression of the transgene (19–21). *ΔiC9* was cloned in-frame, using a *2A*-like sequence from Thosea asigna insect virus (22, 23), with a truncated CD19 domain (*ΔCD19*) serving as a selectable marker to ensure ≥90% purity (20, 24, 25).

The ΔiC9 suicide gene has been investigated in gene modified T cells after allogeneic HSCT (26), CAR T-cells (27), mesenchymal stromal cells (3), iPSC (28), iPSC derived T cells (1) and for cancer therapy (29).

In a phase I clinical trial using the ΔiC9system (21) recipients of CD34-selected haplo-HSCT for hematological malignancies received escalating doses (1x10^6^-1x10^7^ cells/kg) (20, 30) of ΔiC9-modified allo-depleted T cells. In patients with acute graft versus host disease (GVHD) administration of a single dose of 0.4 mg/kg AP1903 resulted in apoptosis of ≥90% of ΔiC9-modified T cells within 30 minutes, followed by a rapid (within 24 hours) and permanent abrogation of GVHD. Remarkably, residual ΔiC9-modified T cells were able to re-expand, contained pathogen-specific precursors, and had a polyclonal T cell receptor repertoire. Although the incomplete elimination can benefit microbiological diseases in the setting of allogeneic HSCT, since the elimination of the gene-modified cells is incomplete [~75-90% of gene-modified T-cells or iPSC (1, 28)], even after repeated (31) or higher doses (32).

Seminal work of Straathof et al. showed 99% elimination of primary T-cells *in vitro* and *in vivo* but only after selection for high transgene expression (19).

As such additional strategies are needed to ensure that the infused, genetically modified cells can be reliably and completely eliminated. Several issues need to be addressed, including (a) improving efficiency of cell killing, (b) predicting inter-individual variations in responsiveness and/or acquisition of resistance, and (c) titrating the therapeutic effect based upon the degree of toxicity., .

Ideally, the strategies would permit flexibility in which the cell therapy could be either downregulated to control moderate toxicities or eliminated completely in the case of severe toxicities.

Our previous finding that the proportion of cells that were not susceptible to ΔiC9-induced apoptosis had elevated levels of BCL-2 (32), prompted us to examine whether BCL-2-mediated pathways could interfere with the induction of apoptosis by the ΔiC9 pathways. We found that combined treatment with the BCL-2 inhibitor, ABT-199 and the BB homodimerizer resulted in the complete elimination of ΔiC9 cells *in vitro* (33). These results support the concept that targeting the mitochondrial apoptotic pathways might enhance the efficiency of ΔiC9.

We hypothesized that co-expression of an additional caspase molecule with an alternative (apoptotic mitochondrial pathway activation) and complementary mechanism of action (activation of the executioner caspase 3) would result in superior/complete gene-modified cell elimination. Since caspase 9 activity is inhibited by direct phosphorylation from mitogen proteins, including the Ser196 residue present in ΔiC9 (34), at a greater extent than what was reported for caspase 8 (35), concurrent inducible activations of caspase 8 would contribute to cell killing in a subset of cells in which CASP9 is inhibited (less likelihood of failure). In parallel, we also tested the combination of ΔiC9 and the RQR8 compact suicide gene activatable by rituxan (36).

Our studies provide fundamental information on the degree and mechanisms of a novel method of cellular regulation based on the ability to activate one or both arms of a combinatorial suicide gene strategy.

## Materials and methods

### Tumor cell lines

All cell lines were freshly acquired from the American Type Culture Collection *(ATCC)*. The Institutional Review Board of the University of Alabama for Human Use at Birmingham (Birmingham, AL) approved this study (IRB# 160516007). 293T HEK17 cells were maintained in culture with DMEM medium *(Thermo Fisher Scientific)* containing 10% fetal bovine serum (FBS) *(GE Healthcare Life Sciences)* and 2mM L-glutamine *(Thermo Fisher Scientific)*. Jurkat clone E6.1 cells were maintained in culture with RPMI 1640 *(GE Healthcare Life Sciences)*, supplemented with 10% FBS and 2 mM L-glutamine.

### Transgene constructs

The nucleotide and amino acid sequence of the constructs employed in this study is reported in the supplementary materials. The *F36V (FKBP12 mutated)* inducible *caspase 9* retroviral vector with a *Δ*CD19 selectable marker was obtained from Baylor College of Medicine (MTA#8733). The *F36V* inducible caspase 8 (*caspase 8* without *CARD* domain) was cloned by PCR and ligated into the SFG vector (gift of Dr. J Maher, King’s College of London) using the Infusion technique *(Takara)*. The SFG-RQR8-IRES-GFP vector was provided by Dr. M. Pule (University College of London) and combined with the inducible caspase 9 by Infusion technique to create SFG.iC9-RQR8-IRES-GFP. The lentiviral constructs *(Elafin alpha promoter, F36V-ΔiC8-T2A-RQR8, and Human PGK promoter FRB-L-FKBP-L-ΔiC9-T2A-ΔCD19)* were cloned by Vectorbuilder. The FKBP12-GGSGG-FKBP12-rapamycin binding domain (FRB)-SGGGSG domains were connected to caspase 9 without CARD (catalytic subunity). We used the same number of amino acid published by Stavrou et al. (37) with a slightly different linker sequence.

The integrity of cloning for all constructs used in this manuscript was confirmed by Sanger sequencing performed either by Vectorbuilder or by the Heflin Center for Human Genetics of the University of Alabama at Birmingham, using the BigDye Terminator v3.1 Cycle Sequencing Ready Reaction kit as per the manufacturer’s instructions (Applied Biosystems). The sequencing products were run following standard protocols on an Applied Biosystems 3730 Genetic Analyzer with POP-7 polymer.

### Transduction

For transduction, replication-incompetent retroviral or lentiviral supernatant was prepared by transfecting *293T* with DNA encoding our construct of interest, the *Peg-Pam-e* plasmid containing the sequence for *MoMLV gag-pol* (or PsPAX2, from Addgene) and the plasmid containing the sequence for the VSVG envelope (Addgene), as previously described (38). The lentiviral supernatant was manufactured by Vectorbuilder or in house using the LV-max third generation packaging system (VSVG envelope). Supernatant harvested at 48 or 72 hours post-transfection was used for transduction. Cells were gene-modified with 2mL of unconcentrated retroviral supernatant on retronectin coated plates for 3 days or overnight with lentiviral supernatant at an MOI of 10, based on ELISA titration, in the presence of Polybrene 16 ug/mL.

### Phenotype

Monoclonal antibodies conjugated with a fluorescent marker were used for flow cytometry as indicated (*BD Biosciences and Invitrogen)*. Expression of the QBend10 selectable marker was assessed using a biotinylated anti-QBend10 monoclonal antibody (Invitrogen) followed by Streptavidine-APC (Biolegend). Cells were analyzed by a FACS Canto II *(BD Biosciences)* for fluorescence signals. For each sample, a minimum of 10,000 viable events were acquired and analyzed using the Kaluza software v.2.1 *(Beckman Coulter: Brea, CA).*

### Killing assay

We performed ***in vitro*** experiments to demonstrate drug elimination of suicide gene-modified cells through activation of the suicide gene of interest. The following drugs were applied at the indicated concentrations unless otherwise stated: non-therapeutic chemical inducer of dimerization, BB homodimerizer, [100nM] *(AP-20187, Clontech; Mountain View, CA)*, sirolimus 25 ng/mL, or rituxan 100 ug/mL in the presence of rabbit serum complement *(Innovative research, Novi, MI).* Cells were incubated overnight with chemical inducer, except in experiments using rituxan, where the incubation time was 4 hours. After the appropriate treatment, cells were washed and stained for viability and apoptosis using the Annexin V/7AAD kits (BD Biosciences). We added 123e counting beads (Invitrogen) and acquired a constant number of beads for each experimental condition using the FACS Canto II. The degree of cellular’s elimination was estimated using the following formula: [*100% - (%Viability treated/% Viability non-treated cells)].* To confirm that killing was due to apoptosis, we performed some experiments after pretreatment with 20 uM of the pan-caspase inhibitor Z-VAD-FMK for 1 hour (BD Pharmingen).

### Regrowth experiments

After the appropriate treatment, cells were washed and re-cultured for regrowth. This was followed by Annexin V/7-AAD staining *(BD Biosciences)* and FACS analysis, as previously indicated.

### Mitochondrial dysfunction

Mytosox red (Invitrogen) was used to test for mitochondrial dysfunction. Cells were treated with 1 ul of a 5 uM working solution. After 10 minutes of incubation at 37° Ccells were washed three times in PBS by centrifugation, followed by flow cytometry acquisition and analysis on FACS Canto II.

### Western blot

Western blot was performed on whole-cell lysates lysed with 1x lysis buffer (Phosphosolutions), NuPage (Invitrogen), a protease inhibitor (Bimake), 10 mM sodium fluoride and 20 mM beta-glycerophosphate (Fisher scientific). An equal number of cells was plated for each condition, and the cells were harvested after 3 hours of incubation. The lysates were separated by electrophoresis using a standard lab technique and transferred using the dry transfer iBlot2 *(Invitrogen).* All primary antibodies (Cell Signaling or Proteintech for GAPDH) were used at 1:1000 dilution and incubated overnight at 4° C. All secondary horseradish peroxidase-conjugated antibodies (Cell signaling rabbit and Proteintech mouse) were used at 1:10,000 and incubated for 1 hour at room temperature. Signal development was performed using the ECL Select detection system (Amersham) and acquired on the G-box automated dark room (Syngene). PageRuler prestained protein ladder was used (Thermofisher).

### Statistical analysis

All data are presented as the average± standard deviation (SD) or standard error of the mean (SEM) where indicated. Unpaired Student *t*-test was used to determine the statistical significance of differences between samples, and a (two-sided) *P-*value less than 0.05 was accepted as a statistically significant difference. Average ± standard error of the mean (SEM) is shown/reported for all the experiments unless otherwise indicated. Data were analyzed and plotted using GraphPad Prism.

## Results

### Generation of an inducible caspase 8

**Figure 1:**
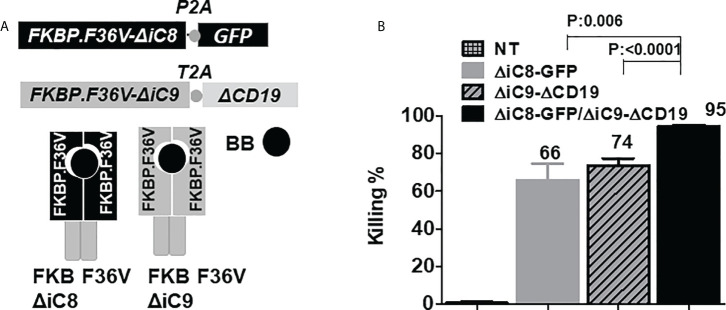
**(A)** *F36V ΔiC8* and *F36V ΔiC9* retroviral constructs and proteins diagram, activatable by a BB homodimerizer. **(B)** Inducible elimination of ΔiC8-GFP, ΔiC9-ΔCD19, and ΔiC8+ΔiC9 co-expressing Jurkat cells (N=5-7 experiments) after overnight exposure to the BB homodimerizer at a concentration of 100 nM.

We generated an inducible caspase 8 (ΔiC8) construct activatable by the BB homodimerizer. ΔiC8 consists of the pro-domains of caspase 8 with or without the caspase activator recruitment domain in frame with the F36V drug-binding domain (**Figure 1A**). After transducing Jurkat cells with replication-incompetent retroviral supernatant, triggering of the inducible caspase 8 after administration of the BB homodimerizer resulted in appreciable cell killing (~20%, not shown); such killing was increased after removal of the endogenous caspase activator recruitment domain (**Figure 1B**, and **Suppl. Table 1**), as previously published (39). Considering these results and that the caspase 8 is upstream of the apoptotic pathway activating both the extrinsic and intrinsic cascade, it was chosen for further evaluation.

### Co-activation of inducible caspase 8 and 9 activated by a homodimerizer

To assess whether co-activation of ΔiC8 and ΔiC9 would result in superior cell elimination compared with the activation of each caspase alone, we performed co-transduction experiments in Jurkat cells transduced with replication-incompetent retroviral supernatant encoding each caspase separately or in combination. ΔiC8 was co-expressed with a GFP selectable marker, and ΔiC9 with a truncated CD19 selectable marker. After overnight exposure, we found that the BB homodimerizer at a concentration of 100nM resulted in the elimination of 66 ± 8.5% ΔiC8-GFP expressing cells, 74 ± 3.5% ΔiC9-ΔCD19 expressing cells, and 95 ± 0.7% ΔiC8+ΔiC9 co-expressing cells, with a statistically significant difference as compared with each caspase alone (P=0.006, or <0.0001, respectively). As a control, exposure of non-transduced (NT) Jurkat to the BB homodimerizer resulted in only 0.7 ± 0.7% killing. N=5-7 experiments (**Figure 1B**).

### Co-activation of inducible caspase 8 and 9 activated by a homodimerizer and a rapalog analog

To activate each caspase with an independent agent, we cloned the FKBP-linker-FRB-linker heterodimerization domain before the inducible caspase 9, enabling dimerization after exposure to the commercially available rapamycin analog sirolimus. Administration of sirolimus results in binding of the pockets with heterodimerization of the FKBP12-rapamycin binding domain (FRB) fragment of mammalian target of rapamycin (mTOR) with FKBP12 and activation of the caspase 9 pathway.

To make our system applicable to different cell types, we used a lentiviral vector system, and either the EF1alpha promoter for the ΔiC8 or the human PGK promoter for the ΔiC9. In those constructs, a truncated human CD19 molecule was again co-expressed with ΔiC9 as a selectable marker. In contrast, for ΔiC8, we used the RQR8 compact selectable marker, which can be detected using the QBend10 CD34 antibody.

**Figure 2:**
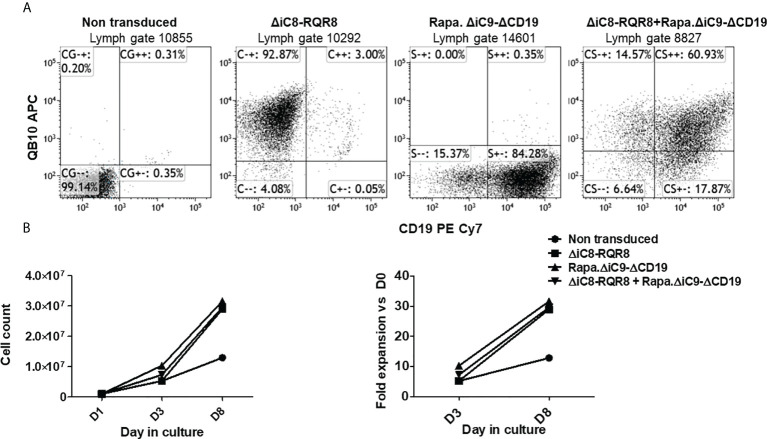
**(A)** Representative flow cytometry expression of the selectable marker for Jurkat non-transduced or gene-modified with (*F36V).ΔiC8-RQR8*, Rapa.*ΔiC9-ΔCD19* or co-transduced. **(B)** (left) Average with SD of cell count and (right) fold expansion of non-transduced or suicide gene-modified cells (one experiment in duplicate).

Jurkat cells were gene-modified with replication-incompetent lentiviral supernatant encoding each construct alone or together as co-transduction. We used an MOI of 10 based on ELISA titration. Jurkat cells gene-modified with the ΔiC8-RQR8 encoding supernatant had 91 ± 1.7% expression of QBEND10. Jurkat cells gene-modified with the Rapa.ΔiC9-ΔCD19 encoding supernatant had 83 ± 1% expression of ΔCD19. Jurkat cells were gene-modified with supernatant encoding the ΔiC8-RQR8, and the Rapa.ΔiC9-ΔCD19 had 56 ± 4% co-expression of QBend10 and ΔCD19. **Figure 2A** shows dot plots from a representative experiment.

**Figure 3:**
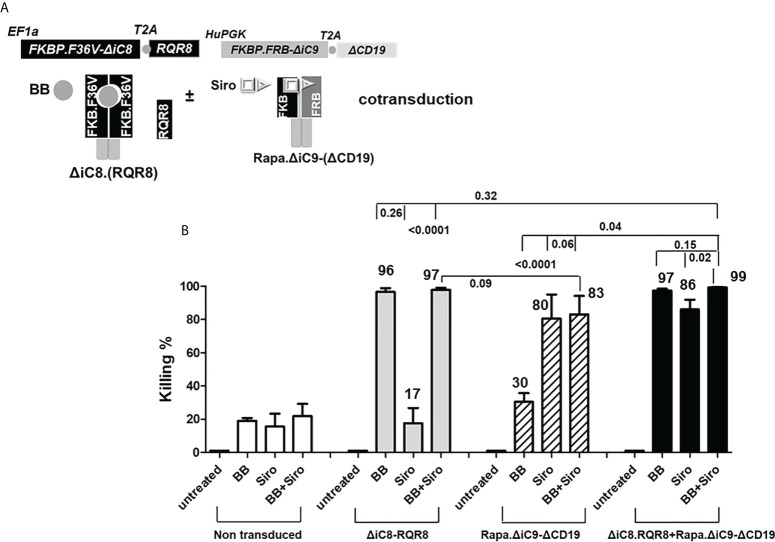
**(A)** *ΔiC8-RQR8* (Elafin1a promoter), Rapa.*ΔiC9-ΔCD19* (Human-PGK promoter) lentiviral constructs, and proteins diagram; *ΔiC8 and* Rapa.*ΔiC9* are activatable by the BB homodimerizer or sirolimus, respectively. **(B)** Average with SD of the percentage of killing in non-transduced Jurkat cells, or Jurkat cells gene-modified to express ΔiC8-RQR8, Rapa.ΔiC9-ΔCD19, or both (N: 3-7 experiments) after overnight exposure to the BB homodimerizer at 100nM, sirolimus (siro) 25 ng/mL or both.

Cells were not enriched for transgene expression using selection because (i) the expression of the selectable marker was robust, and (ii) non-gene-modified cells act as an internal control after killing. Cells were able to expand in culture (**Figure 2B**) despite the constitutive expression of a suicide gene, and in this limited observation gene-modified cells expanded at a higher rate as compared with non-transduced cells. In standard killing experiments, we used the BB homodimerizer at a concentration of 100 nM, which was previously demonstrated as a plateau concentration *in vitro*, and readily achievable in patients after therapeutic dosing. We used sirolimus at the therapeutic concentration of 25 ng/mL. We observed ~20% background cell elimination in non-transduced cells treated with the BB homodimerizer or sirolimus. Activation of each construct alone resulted in elimination through apoptosis of a significant number of gene-modified cells (≥80%). Notably, there was no interference between the two systems (**Figures 3A, B**).

For the cells co-transduced with both constructs, the percentage of cell killing was statistically higher (P=0.04) for the inducible ΔiC8 (97 ± 1%) than Rapa.ΔiC9 (86 ± 6%).

Notably, the cell-killing observed using a single agent in cells co-transduced with both constructs was comparable to cells transduced with a single construct. The combination of the two suicide genes resulted in higher elimination of cells (99 ± 0.5%), which was statistically significant as compared with sirolimus alone (P=0.02), and biologically relevant as compared with BB homodimerizer alone (**Figures 3A, B** and **Suppl. Table 2**). For regrowth experiments, cells were recultured an additional 14 days, then recounted. We found that the number of ΔiC8-RQR8/Rapa.ΔiC9 suicide gene-modified cells regrowing in the condition treated with both agents was statistically significantly lower and at the background level in all experiments. The average cell count and standard error for the untreated condition, was 2.4e6 ± 3.2e4, for the BB treated condition was 1.5e6 ± 1.2e4, for the sirolimus treated condition was 1e6± 2.3e4, and for the BB/sirolimus treated condition, 6.5e4 ± 3.1e3 (**Figure 5B**). The superior reduction in cell regrowth after activating both systems is potentially linked to preventing resistance mechanisms (**Figure 5B**).

**Figure 4:**
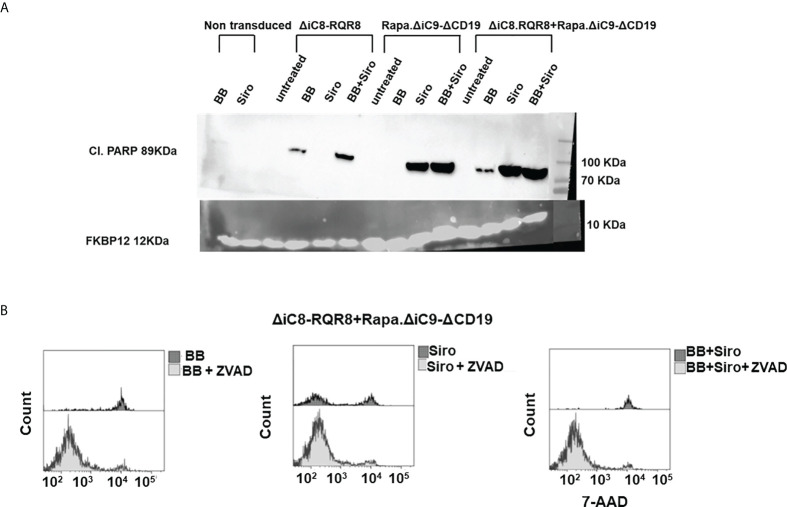
**(A)** Western blot for cleavage of the effector of the apoptosis Poly (ADP-ribose) polymerase (PARP), or FKBP12 endogenous control. **(B)** Histogram depicting viable ΔiC8-RQR8/Rapa.ΔiC9-ΔCD19 Jurkat cells after administering the BB homodimerizer, sirolimus (siro) or both in the absence or presence of the ZVAD fmk pan-caspases inhibitor.

Cell elimination was preceded by the cleavage of the effector of the apoptosis Poly (ADP-ribose) polymerase (PARP). Consistent with the lack of interference between the two systems, as shown in the cell elimination assay, only the specific activation of each construct with the appropriate agent resulted in cleavage of PARP (**Figure 4A**).

**Figure 5:**
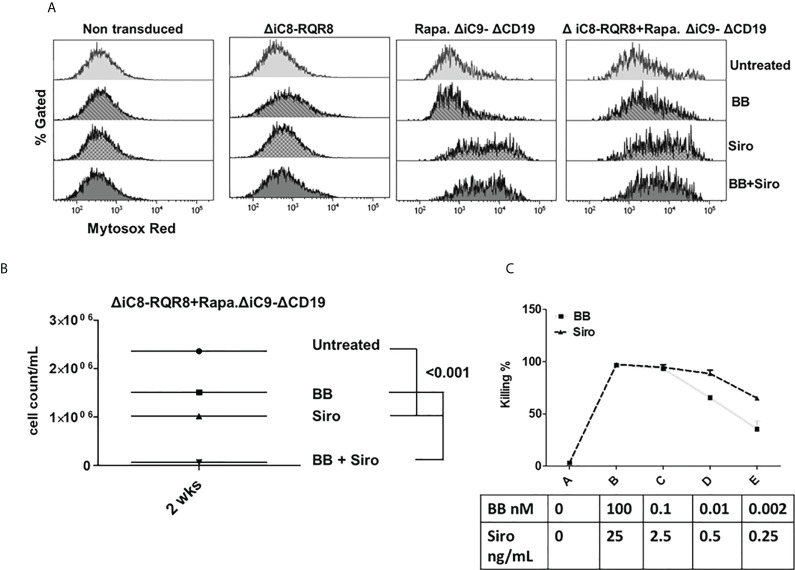
**(A)** Mitochondrial dysfunction assessed with the MytoSox Red flow cytometry method on ΔiC8-RQR8, Rapa.ΔiC9a-ΔCD19, or F36VΔiC8/RapaΔiC9 co-expressing cells treated with the BB homodimerizer, sirolimus (siro) or both. **(B)**: Day 14 analysis of regrowth (N: 3) of ΔiC8-RQR8/Rapa.ΔiC9 suicide gene-modified Jurkat cells treated with the BB homodimerizer, sirolimus (siro) or both. **(C)** Titration of ΔiC8-RQR8/Rapa.ΔiC9 suicide gene-modified Jurkat cells with decreasing concentration of BB homodimerizer or sirolimus.

Mitochondrial dysfunction was assessed using the MytoSox Red flow cytometry method (**Figure 5A**). Interestingly, the magnitude of mitochondrial dysfunction was higher with sirolimus-treated cells. The cell elimination was reversed by pre-treatment with a pan-caspases inhibitor (**Figure 4B**).

We report the expression of the selectable marker on day 1 and on day 14 after the killing assay and reculturing the cells *in vitro* from one representative experiment (**Figure S1**).

Titration experiments showed that the BB homodimerizer offered the flexibility of titration in doses potentially achievable in the clinical setting. Sirolimus remained very potent at minimal concentrations unachievable *in vivo* (**Figure 5C**).

### Co-expression of inducible caspase 9 and the RQR8 compact suicide gene

**Figure 6:**
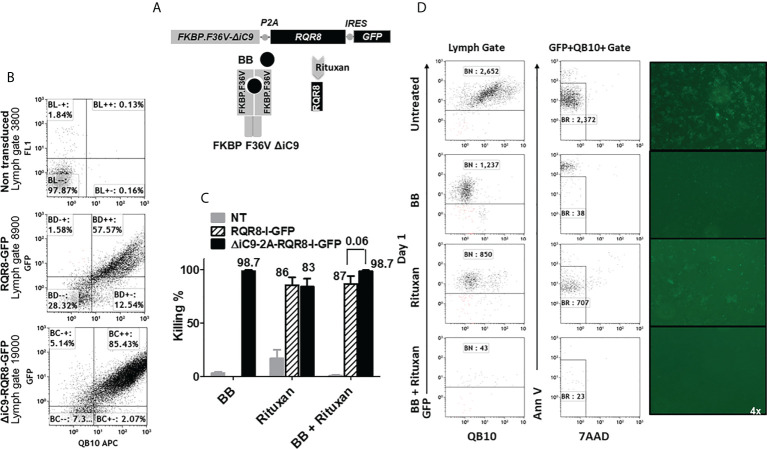
**(A)** Retroviral transgenic construct and protein diagram of *ΔiC9-RQR8-GFP* activatable with the BB homodimerizer and the anti-CD20 monoclonal antibody rituxan. 6**(B)** Dot-plots of QBend10/GFP expression from a representative experiment. **(C)** Summary of N: 3-5 killing experiments of Jurkat non-transduced, expressing the RQR8 compact suicide gene or the iC9 and the RQR8 suicide gene in a single construct treated with the BB homodimerizer overnight [100nM], or rituxan [100 ug/mL] for four hours in the presence of rabbit serum complement. **(D)**. Dot-plot and fluorescent images from one representative killing assay.

In parallel, we investigated if expressing two suicide genes with a different mechanism of action would lead to the complete elimination of gene-modified cells. We generated a construct co-expressing the ΔiC9, the RQR8 compact suicide gene, and a GFP selectable marker. In addition to the QBend10 as a selectable marker, the RQR8 compact suicide gene contains a CD20 mimotope that the CD20 antibody that rituxan can target, resulting in complement and antibody-dependent cytotoxicity, **Figure 6A**. We gene-modified Jurkat cells to express the RQR8-GFP transgene alone or with the ΔiC9 in a single construct (ΔiC9-RQR8-GFP).

We assessed the co-expression of the GFP and the RQR8 by flow cytometry. Non-transduced cells had 0.13% ± 0 co-expression, RQR8-GFP 81% ± 11, and ΔiC9-RQR8-GFP 82% ± 2.8 (N: 2), as shown in **Figure 6B** and **Suppl. Table 3**.

The ΔiC9 suicide gene alone resulted in 98% cell elimination after applying the BB dimerizer. As expected, the killing with rituxan or the BB dimerizer of non-transduced cells was negligible. Rituxan alone resulted in 80 ± 11% elimination of gene-modified cells, whereas sequential administration of the BB dimerizer followed by rituxan resulted in 98 ± 1% elimination (P=0.06). The co-activation of the ΔiC9 and RQR8 suicide genes improved killing compared to that achieved with activation of the RQR8 suicide gene alone with rituxan (**Figures 6C, D**), with complete elimination of the gene-modified cells with no regrowth (not shown). 

## Discussion

Our studies provide information on the activity of two suicide gene systems expressed in combination for the safety of cellular therapeutics. Our first system is based on an inducible caspase 8, activatable with a BB homodimerizer, and an inducible caspase 9, activatable after the administration of sirolimus

The second system is based on an inducible caspase 9 activatable by the BB homodimerizer and the RQR8 compact suicide gene, targeted by an anti-CD20 monoclonal antibody, such as rituxan, Both the RQR8 and the Rapamycin-activatable caspase 9 have been investigated *in vitro* and *in vivo* in mice in combination with CAR T-cells targeting CD19 (36, 37) and proved effective in inducing cell elimination. We observed a superior cell killing after using the homodimerizer to activate either caspase 8 or caspase 9, compared with the activation of Rapa.ΔiC9 with sirolimus. Differences in vector design, including length and structure of the linker sequence separating the two domains of the Rapa.ΔiC9, may account for such differences as previously reported (37). Our experiments estimated the degree of cell killing for each system independently or in combination, achieving a more comprehensive cell elimination when activating both arms of either system. The superior reduction in cell regrowth after activating both systems is potentially linked to preventing resistance mechanisms. The results add to the literature on combination suicide gene systems. Shah et al. (40) published on a mifepristone-induced gene expression of inducible caspase 3 and inducible caspase 9 activatable by the BB homodimerizer *in vitro* and a novel mice model. However, in this study, a quantification of cell killing with each suicide gene alone or in combination is missing. Fang et al. (41) developed a reversibly immortalized hepatic progenitor cell line for regenerative medicine, where the removal of the SV40T gene was guaranteed through HSV-TK/GCV selection. In contrast, cell elimination was performed under the control of a single cytosine deamines/5-fluoruracil suicide gene system. In addition to the limitation of using metabolic suicide gene systems, it is unclear whether cells could regrow after activating the suicide gene. Martin et al. (42) published a study on improving the safety of iPSC using genome-edited orthogonal safeguard. They used targeted integration to express an inducible caspase 9 activated by the BB homodimerizer at the end of the NANOG gene using a 2A sequence, showing the ability to prevent or ablate teratomas. They integrated a second suicide gene after the ACTB gene using a 2A sequence to eliminate differentiated cells. After observing that the HSV-TK system was slow and preferentially killing proliferating cells, they investigated using an inducible caspase 9 activated by the AC heterodimerizer. While this system is similar to a sirolimus-induced safety switch, the AC heterodimerizer has never been investigated in patients and is not available for clinical infusion. This study supports using two safety switches, albeit here each for a different cellular differentiation state. Based on the risk of incomplete cell elimination with a single suicide gene, a double system for each differentiation state is more ideal. Since selectable markers are useful for cell selection or tracking, incorporating RQR8 would also grant an additional safety measure. Additionally, using two different caspases would reduce the risk of gene recombination.

It is crucial to perform safety studies to elucidate the effect on gene-modified cells, such as the propensity for insertional mutagenesis and tumorigenesis in stem cell products. Albeit chromosomal position effects are less likely to silence two suicide switches provided on two independent vectors, we plan to compare them with a single construct to assess if it can already circumvent this effect in case of high transgene copy numbers, as previously reported (19).

The inducible Caspase 9 (43) and the RQR8 suicide gene are under active clinical investigation in CAR-T clinical trials. In a case report, activating the inducible caspase 9 with the BB homodimerizer rimiducid resulted in the resolution of ICANS that was resistant to the administration of tocilizumab and corticosteroids (27). In a subsequent small clinical trial in nine patients, the infusion of iC9-CAR-T targeting CD19 proved safe and effective in controlling leukemia. However, none of the patients met the eligibility criteria to activate the suicide gene (43). The authors are also investigating if a lower dose of rimiducid would ameliorate the CRS/ICANS without eliminating the infused CAR-T cells (43).

One alternative approach would be to combine a suicide genes with other strategies to mitigate side effects from the infused cells, activating the suicide gene only as a last resort.

Wiebking et al. published on a transgene-free safety switch in cell lines, pluripotent cells and primary human T-cells. Using genome editing methods, they disrupted uridine monophosphate synthetase in the pyrimidine *de novo* synthesis pathway making proliferation dependent on external uridine and enabling to control cell growth by modulating the uridine supply, both *in vitro* and *in vivo* in a murine model. Additionally, disrupting this pathway created resistance to 5-fluoroorotic acid, enabling positive selection of the gene edited cells (44).

Other strategies are more specific for CAR T-cells include logic-gated CAR T-cells with functional operations triggered by two input signals. For example, it is possible to increase the specificity by either requiring recognition of two antigens on the cell’s surface (bispecific CAR) or the absence of an antigen (inhibitory CAR). An additional approach involves the use of a modular CAR which is split into two interactive parts, the signaling module on T cells and the switching module is administered separately to recognize the target antigen. The use of T-cells electroporated with mRNA encoding a CAR molecule granted transient CAR expression, providing an additional method for finer spatial and temporal control. Majzner et al. (45) found that change in signaling domains or the hinge-transmembrane domain region can alter activity against low vs. high antigen tumors to open a therapeutic window that could prevent possible on-target off-tumor toxicity.

A regulatable elimination of cellular therapeutics is also relevant in allogeneic HSCT. It is well accepted that allogeneic T-cells mediate both GVHD and a graft-versus-tumor effect (46). In the setting of GVHD, it was hypothesized that high levels of transcription of the ΔiC9 transgene caused by T-cell-receptor activation in alloreactive T-cells explained the selective elimination of these cells by the dimerizer (21), and it is important to experiment if a low dose of dimerizer would control of graft-versus-host disease while maintaining a few alloreactive cells potentially granting a graft-versus-tumor effect.

While the incomplete elimination proved of some benefit to control microbiological disease after allogeneic HSCT (21), it is necessary to completely eliminate the infused gene-modified cells for severe toxicities. The reason why some cells survived after activation of the inducible caspase 9 remains elusive. Hypotheses include the survival of cells with a low level of transgene expression or with higher expression of anti-apoptotic molecules. Chang *et al.* reported that the elimination of ΔiC9 gene-modified cells is determined by a minimum expression threshold of the transgene in activated T-cells, which is dependent on T-cell receptor activation state of the T-cells, as well as *cis*-acting influences by host promoters on the proviral transgene (47).

Our approaches also offer a model to study a specific inducible cell death pathway by inducing caspase 8 or 9 in this case. Building on this, we are also investigating the combination of an inducible caspase with inducible strategies to inactivate anti-apoptotic molecules (e.g. BCL-2) or to induce an additional pro-apoptotic molecule.

Furthermore, the results of our study have applications beyond CAR T-cells and support the development of a cellular safety switch for genetically modified stem cells and other iPSC-derived progeny for cancer or regenerative medicine. There is growing interest in generating off-the-shelf T-cell therapy products for treating solid cancer or hematologic malignancies, which is important for patients with quantitative or qualitative T-cells defects (48). Additionally, applying gene-editing techniques aimed at knocking out the endogenous T-cell receptor (49, 50) and/or human leukocyte antigens (HLA) molecules (51) is essential to reduce alloreactivity and immune response, enabling the infusion of cellular therapeutics across HLA barriers. However, with the concern of insertional mutagenesis, the incorporation of a suicide gene is ideal. Examples of insertional mutagenesis and malignant transformation were reported in gene-modified HSC using gamma retroviral vectors in clinical trials for severe combined immune deficiency (6–8), X-linked chronic granulomatous disease (52), and Wiskott-Aldrich syndrome (10). Clonal dominance has also been reported from a clinical trial in beta-thalassemia using a lentiviral vector (9, 14). Modifications of the lentiviral vector (incorporation of insulator sequences) have reduced some complications, and this approach is currently under clinical investigation in patients with hemoglobinopathies (NCT01745120 and NCT02140554), and sickle cell disease (NCT04293185). Integration hotspots have been identified in stem cell products of some patients without transformation events.

In conclusion, we performed *in vitro* validation of two inducible suicide gene combinations (caspase 8: caspase 9 and caspase 9:RQR8). We showed that gene-modifying cells with two suicide gene constructs and a selectable marker is feasible. We also demonstrate that the two systems can be activated independently to control the cells of interest, with superior cell killing with superior reduction in cell regrowth compared to single suicide gene systems. While results need to be confirmed in other cell types, especially primary cells, they provide a framework for enhancing the safety of cellular therapeutics, facilitating the translation of novel gene therapy strategies in the pre-clinical and clinical setting.

## Data availability statement

The original contributions presented in the study are included in the article/**Supplementary Materials**. Further inquiries can be directed to the corresponding author.

## Author contributions

CF and LS performed cloning and experiments, drafted the manuscript, edited, and reviewed the final version of the manuscript. MA-O, MA-Z, and KP performed cloning and experiments, edited, and reviewed the final version of the manuscript. KM, contributed to vector’s cloning, edited, and reviewed the final version of the manuscript. MA, DS, RB, and FG, concept design, monitoring, reviewed data, and reviewed the final version of the manuscript. AD conceived the idea, designed the experiments, supervised research, analyzed the results, reviewed, and edited the final version of the manuscript. All authors contributed to the article and approved the submitted version.

## Funding

St. Baldrick’s Foundation, Post-Doctoral Fellowship for Childhood Cancer Research, Dixon Award Scholarship.

## Conflict of interest

The authors declare that the research was conducted in the absence of any commercial or financial relationships that could be construed as a potential conflict of interest.

## Publisher’s note

All claims expressed in this article are solely those of the authors and do not necessarily represent those of their affiliated organizations, or those of the publisher, the editors and the reviewers. Any product that may be evaluated in this article, or claim that may be made by its manufacturer, is not guaranteed or endorsed by the publisher.

## Supplementary material

Supplementary Figure 1 Expression of the selectable marker on days 1 and 14 after killing from one representative experiment using Jurkat expressing ΔiC8-RQR8, Rapa.ΔiC9- ΔCD19 or both, treated with the BB homodimerizer [100nM], sirolimus (siro) [25 ng/mL] or both.
