# Supplementary figures and images for: Combinatorial suicide gene strategies for the safety of cell therapies
Source: Front Immunol. 2022 Sep 14;13:975233. doi: 10.3389/fimmu.2022.975233 (PMC9515659; doi:10.3389/fimmu.2022.975233)

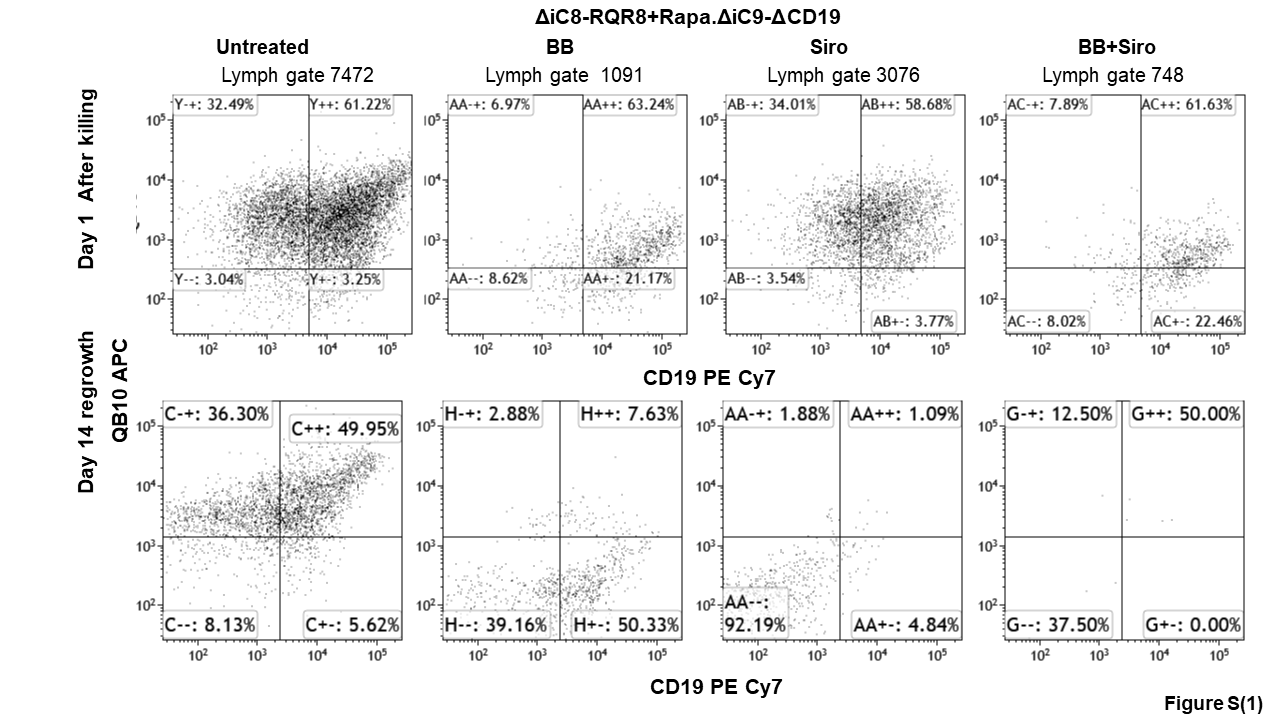

Supplement: Supplementary Figure 1 — Expression of the selectable marker on days 1 and 14 after killing from one representative experiment using Jurkat expressing ΔiC8-RQR8, Rapa.ΔiC9- ΔCD19 or both, treated with the BB homodimerizer [100nM], sirolimus (siro) [25 ng/mL] or both. [file Image_1.tif]

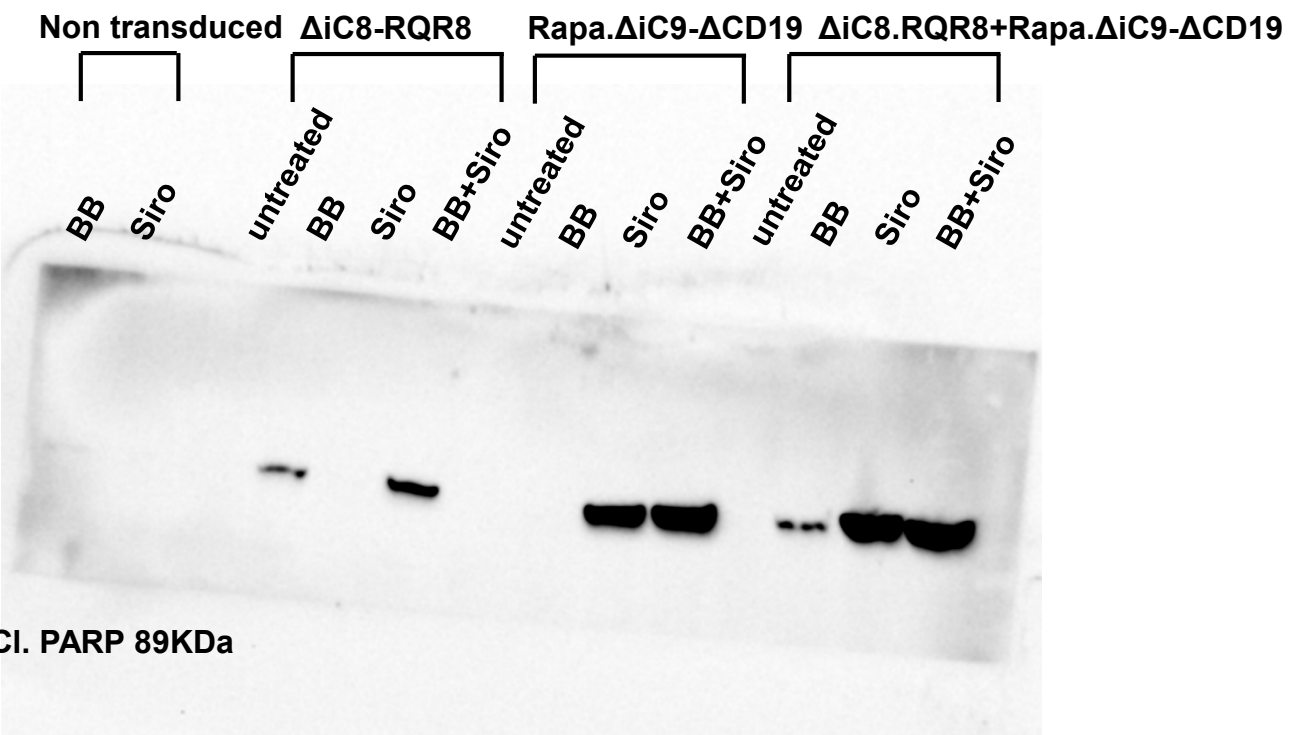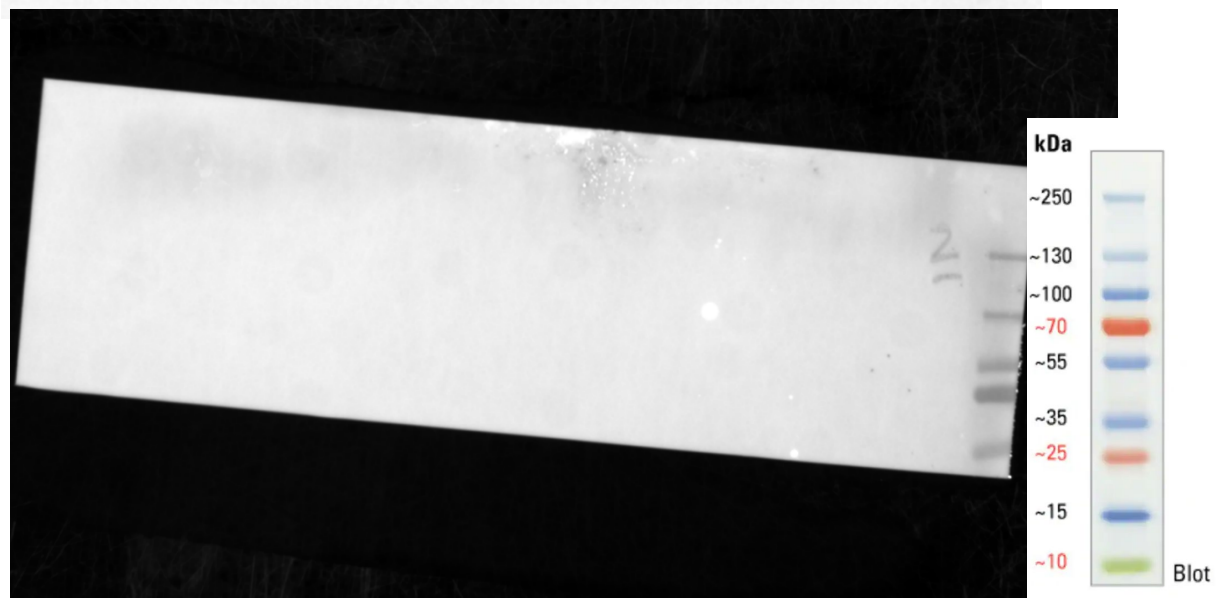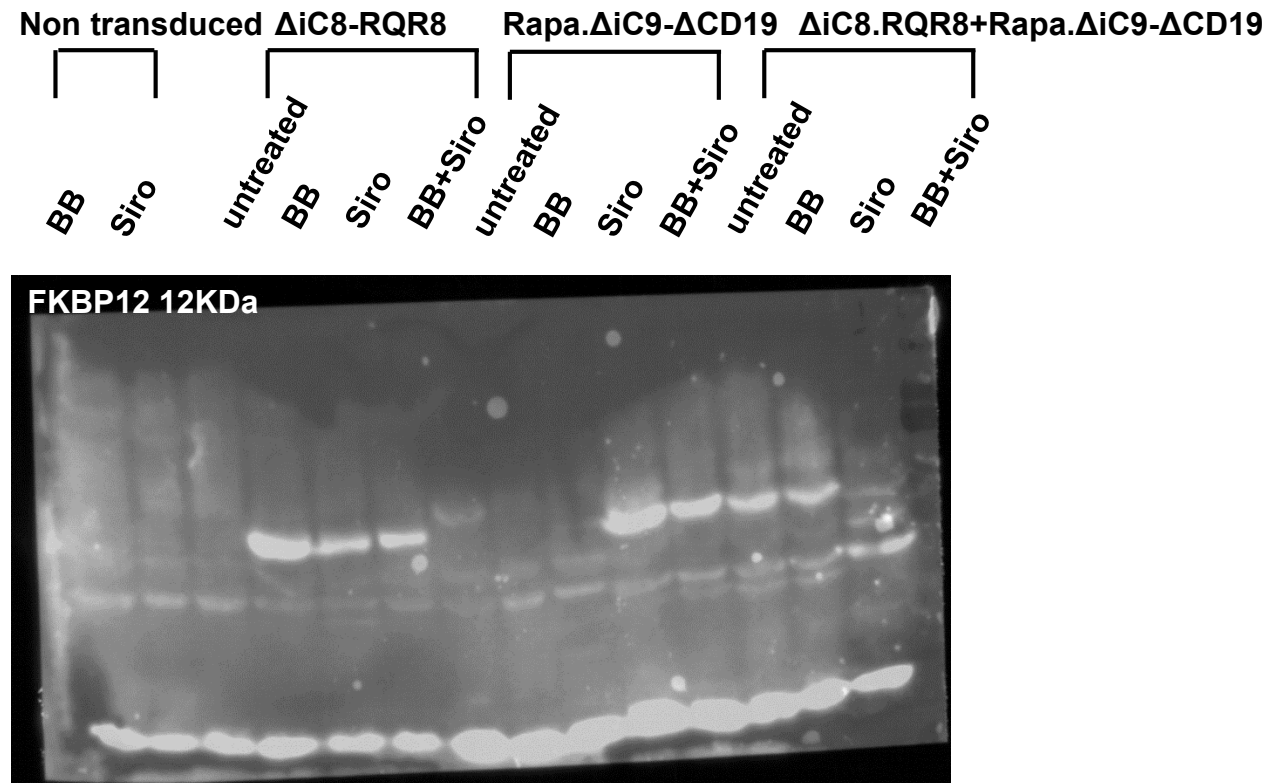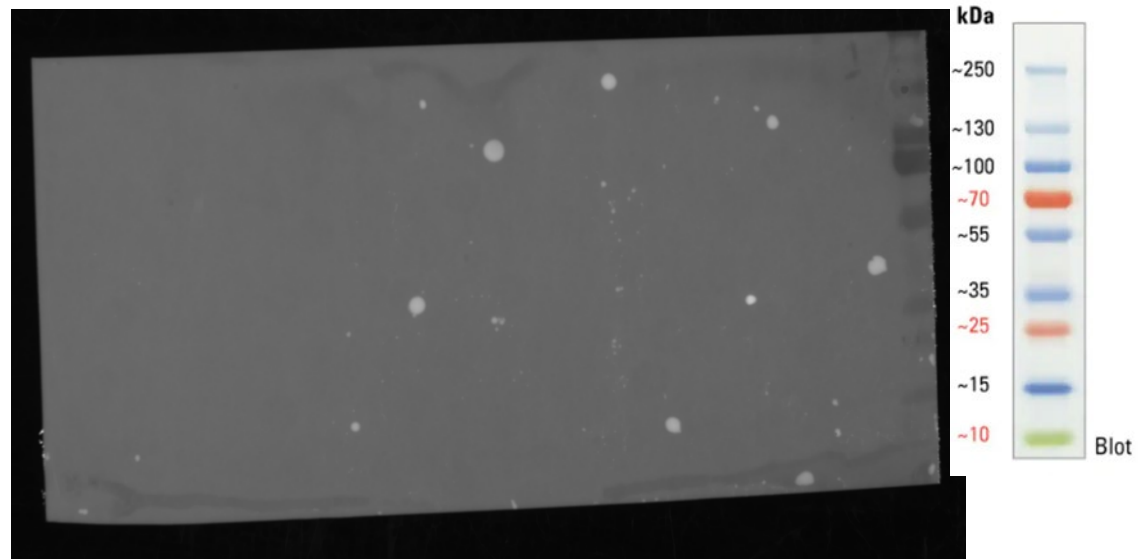

Supplement: Supplementary file 3 [file DataSheet_2.pdf]
