# Supplementary material for: Combinatorial suicide gene strategies for the safety of cell therapies
Source: Front Immunol. 2022 Sep 14;13:975233. doi: 10.3389/fimmu.2022.975233 (PMC9515659; doi:10.3389/fimmu.2022.975233)
Supplement: Supplementary file 2 [file DataSheet_1.pdf]

DNA and AMINO ACID SEQUENCES

>F36V-ΔCaspase8-P2A-GFP

ATGCTCGAGGGAGTGCAGGTGGAAACCATCTCCCCAGGAGACGGGCGCACCTTCCCCAAGCGCGGCCAGACCTG
CGTGGTGCACTACACCGGGATGCTTGAAGATGGAAAGAAAGTTGATTCCTCCCGGGACAGAAACAAGCCCTTTAA
GTTTATGCTAGGCAAGCAGGAGGTGATCCGAGGCTGGGAAGAAGGGGTTGCCCAGATGAGTGTGGGTCAGAGA
GCCAAACTGACTATATCTCCAGATTATGCCTATGGTGCCACTGGGCACCCAGGCATCATCCCACCACATGCCACTCT
CGTCTTCGATGTGGAGCTTCTAAAACTGGAATCTGGCGGTGGATCCacaatctcggactctccaagagaacaggatagtgaatc
acagactttggacaaagtttaccaaatgaaaagcaaacctcggggatactgtctgatcatcaacaatcacaattttgcaaaagcacgggagaaagtg
cccaaacttcacagcattagggacaggaatggaacacacttggatgcaggggctttgaccacgacctttgaagagcttcattttgagatcaagcccca
cgatgactgcacagtagagcaaatctatgagattttgaaaatctaccaactcatggaccacagtaacatggactgcttcatctgctgtatcctctcccat
ggagacaagggcatcatctatggcactgatggacaggaggcccccatctatgagctgacatctcagttcactggtttgaagtgcccttcccttgctgga
aaacccaaagtgtttttattcaggcttgtcaggggataactaccagaaaggtatacctgttgagactgattcagaggagcaaccctatttagaaatg
gatttatcatcacctcaaacgagatatatcccggatgaggctgactttctgctggggatggccactgtgaataactgtgtttcctaccgaaaccctgcag
agggaacctggtacatccagtcactttgccagagcctgagagagcgatgtcctcgaggcgatgatattctcaccatcctgactgaagtgaactatgaa
gtaagcaacaaggatgacaagaaaaacatggggaaacagatgcctcagcctactttcacactaagaaaaaaacttgtcttcccttctgatagcgctG
CAACAAACTTCTCACTACTCAAACAAGCAGGTGACGTGGAGGAGAATCCCGGGCCTAACATGGTGAGCAAGGGC
GAGGAGCTGTTCACCGGGGTGGTGCCCATCCTGGTCGAGCTGGACGGCGACGTAAACGGCCACAAGTTCAGCGT
GTCCGGCGAGGGCGAGGGCGATGCCACCTACGGCAAGCTGACCCTGAAGTTCATCTGCACCACCGGCAAGCTGC
CCGTGCCCTGGCCCACCCTCGTGACCACCCTGACCTACGGCGTGCAGTGCTTCAGCCGCTACCCCGACCACATGAA
GCAGCACGACTTCTTCAAGTCCGCCATGCCCGAAGGCTACGTCCAGGAGCGCACCATCTTCTTCAAGGACGACGG
CAACTACAAGACCCGCGCCGAGGTGAAGTTCGAGGGCGACACCCTGGTGAACCGCATCGAGCTGAAGGGCATCG
ACTTCAAGGAGGACGGCAACATCCTGGGGCACAAGCTGGAGTACAACTACAACAGCCACAACGTCTATATCATGG
CCGACAAGCAGAAGAACGGCATCAAGGTGAACTTCAAGATCCGCCACAACATCGAGGACGGCAGCGTGCAGCTC
GCCGACCACTACCAGCAGAACACCCCCATCGGCGACGGCCCCGTGCTGCTGCCCGACAACCACTACCTGAGCACCC
AGTCCGCCCTGAGCAAAGACCCCAACGAGAAGCGCGATCACATGGTCCTGCTGGAGTTCGTGACCGCCGCCGGG
ATCACTCTCGGCATGGACGAGCTGTACAAGTAA

MLEGVQVETISPGDGRTFPKRGQTCVVHYTGMLEDGKKVDSSRDRNKPFKFMLGKQEVIRGWEEGVAQMSVGQRA
KLTISPDYAYGATGHPGIIPPHATLVFDVELLKLESGGGSTISDSPREQDSESQTLDKVYQMKSKPRGYCLIINNHNFAKAR
EKVPKLHSIRDRNGTHLDAGALTTTFEELHFEIKPHDDCTVEQIYEILKIYQLMDHSNMDCFICCILSHGDKGIIYGTDGQE
APIYELTSQFTGLKCPSLAGKPKVFFIQACQGDNYQKGIPVETDSEEQPYLEMDLSSPQTRYIPDEADFLLGMATVNNCV
SYRNPAEGTWYIQSLCQSLRERCPRGDDILTILTEVNYEVSNKDDKKNMGKQMPQPTFTLRKKLVFPSDSAATNFSLLK
QAGDVEENPGPNMVSKGEELFTGVVPILVELDGDVNGHKFSVSGEGEGDATYGKLTLKFICTTGKLPVPWPTLVTTLTY
GVQCFSRYPDHMKQHDFFKSAMPEGYVQERTIFFKDDGNYKTRAEVKFEGDTLVNRIELKGIDFKEDGNILGHKLEYNY
NSHNVYIMADKQKNGIKVNFKIRHNIEDGSVQLADHYQQNTPIGDGPVLLPDNHYLSTQSALSKDPNEKRDHMVLLEF
VTAAGITLGMDELYK*

>F36V-ΔCaspase9-T2A-ΔCD19

ATGCTCGAGGGAGTGCAGGTGGAAACCATCTCCCCAGGAGACGGGCGCACCTTCCCCAAGCGCGGCCAGACCTG
CGTGGTGCACTACACCGGGATGCTTGAAGATGGAAAGAAAGTTGATTCCTCCCGGGACAGAAACAAGCCCTTTAA
GTTTATGCTAGGCAAGCAGGAGGTGATCCGAGGCTGGGAAGAAGGGGTTGCCCAGATGAGTGTGGGTCAGAGA
GCCAAACTGACTATATCTCCAGATTATGCCTATGGTGCCACTGGGCACCCAGGCATCATCCCACCACATGCCACTCT
CGTCTTCGATGTGGAGCTTCTAAAACTGGAATCTGGCGGTGGATCCGGAGTCGACGGATTTGGTGATGTCGGTGC
TCTTGAGAGTTTGAGGGGAAATGCAGATTTGGCTTACATCCTGAGCATGGAGCCCTGTGGCCACTGCCTCATTATC

AACAATGTGAACTTCTGCCGTGAGTCCGGGCTCCGCACCCGCACTGGCTCCAACATCGACTGTGAGAAGTTGCGG
CGTCGCTTCTCCTCGCTGCATTTCATGGTGGAGGTGAAGGGCGACCTGACTGCCAAGAAAATGGTGCTGGCTTTGC
TGGAGCTGGCGCAGCAGGACCACGGTGCTCTGGACTGCTGCGTGGTGGTCATTCTCTCTCACGGCTGTCAGGCCA
GCCACCTGCAGTTCCCAGGGGCTGTCTACGGCACAGATGGATGCCCTGTGTCGGTCGAGAAGATTGTGAACATCT
TCAATGGGACCAGCTGCCCCAGCCTGGGAGGGAAGCCCAAGCTCTTTTTCATCCAGGCCTGTGGTGGGGAGCAGA
AAGACCATGGGTTTGAGGTGGCCTCCACTTCCCCTGAAGACGAGTCCCCTGGCAGTAACCCCGAGCCAGATGCCA
CCCCGTTCCAGGAAGGTTTGAGGACCTTCGACCAGCTGGACGCCATATCTAGTTTGCCCACACCCAGTGACATCTT
TGTGTCCTACTCTACTTTCCCAGGTTTTGTTTCCTGGAGGGACCCCAAGAGTGGCTCCTGGTACGTTGAGACCCTGG
ACGACATCTTTGAGCAGTGGGCTCACTCTGAAGACCTGCAGTCCCTCCTGCTTAGGGTCGCTAATGCTGTTTCGGT
GAAAGGGATTTATAAACAGATGCCTGGTTGCTTTAATTTCCTCCGGAAAAAACTTTTCTTTAAAACATCAGCTAGCA
GAGCCGAGGGCAGGGGAAGTCTTCTAACATGCGGGGACGTGGAGGAAAATCCCGGGCCCATGCCACCTCCTCGC
CTCCTCTTCTTCCTCCTCTTCCTCACCCCCATGGAAGTCAGGCCCGAGGAACCTCTAGTGGTGAAGGTGGAAGAGG
GAGATAACGCTGTGCTGCAGTGCCTCAAGGGGACCTCAGATGGCCCCACTCAGCAGCTGACCTGGTCTCGGGAGT
CCCCGCTTAAACCCTTCTTAAAACTCAGCCTGGGGCTGCCAGGCCTGGGAATCCACATGAGGCCCCTGGCCATCTG
GCTTTTCATCTTCAACGTCTCTCAACAGATGGGGGGCTTCTACCTGTGCCAGCCGGGGCCCCCCTCTGAGAAGGCC
TGGCAGCCTGGCTGGACAGTCAATGTGGAGGGCAGCGGGGAGCTGTTCCGGTGGAATGTTTCGGACCTAGGTGG
CCTGGGCTGTGGCCTGAAGAACAGGTCCTCAGAGGGCCCCAGCTCCCCTTCCGGGAAGCTCATGAGCCCCAAGCT
GTATGTGTGGGCCAAAGACCGCCCTGAGATCTGGGAGGGAGAGCCTCCGTGTCTCCCACCGAGGGACAGCCTGA
ACCAGAGCCTCAGCCAGGACCTCACCATGGCCCCTGGCTCCACACTCTGGCTGTCCTGTGGGGTACCCCCTGACTC
TGTGTCCAGGGGCCCCCTCTCCTGGACCCATGTGCACCCCAAGGGGCCTAAGTCATTGCTGAGCCTAGAGCTGAA
GGACGATCGCCCGGCCAGAGATATGTGGGTAATGGAGACGGGTCTGTTGTTGCCCCGGGCCACAGCTCAAGACG
CTGGAAAGTATTATTGTCACCGTGGCAACCTGACCATGTCATTCCACCTGGAGATCACTGCTCGGCCAGTACTATG
GCACTGGCTGCTGAGGACTGGTGGCTGGAAGGTCTCAGCTGTGACTTTGGCTTATCTGATCTTCTGCCTGTGTTCC
CTTGTGGGCATTCTTCATCTTCAAAGAGCCCTGGTCCTGAGGAGGAAAAGAAAGCGAATGACTGACCCCACCAGG
AGATTCTAA

MLEGVQVETISPGDGRTFPKRGQTCVVHYTGMLEDGKKVDSSRDRNKPFKFMLGKQEVIRGWEEGVAQMSVGQRA
KLTISPDYAYGATGHPGIIPPHATLVFDVELLKLESGGGSGVDGFGDVGALESLRGNADLAYILSMEPCGHCLIINNVNFC
RESGLRTRTGSNIDCEKLRRRFSSLHFMVEVKGDLTAKKMVLALLELAQQDHGALDCCVVVILSHGCQASHLQFPGAVY
GTDGCPVSVEKIVNIFNGTSCPSLGGKPKLFFIQACGGEQKDHGFEVASTSPEDESPGSNPEPDATPFQEGLRTFDQLDA
ISSLPTPSDIFVSYSTFPGFVSWRDPKSGSWYVETLDDIFEQWAHSEDLQSLLLRVANAVSVKGIYKQMPGCFNFLRKKL
FFKTSASRAEGRGSLLTCGDVEENPGPMPPPRLLFFLLFLTPMEVRPEEPLVVKVEEGDNAVLQCLKGTSDGPTQQLTW
SRESPLKPFLKLSLGLPGLGIHMRPLAIWLFIFNVSQQMGGFYLCQPGPPSEKAWQPGWTVNVEGSGELFRWNVSDLG
GLGCGLKNRSSEGPSSPSGKLMSPKLYVWAKDRPEIWEGEPPCLPPRDSLNQSLSQDLTMAPGSTLWLSCGVPPDSVS
RGPLSWTHVHPKGPKSLLSLELKDDRPARDMWVMETGLLLPRATAQDAGKYYCHRGNLTMSFHLEITARPVLWHWL
LRTGGWKVSAVTLAYLIFCLCSLVGILHLQRALVLRRKRKRMTDPTRRF*

> F36V-ΔCaspase9-T2A-RQR8-I-GFP

ATGCTCGAGGGAGTGCAGGTGGAAACCATCTCCCCAGGAGACGGGCGCACCTTCCCCAAGCGCGGCCAGACCTG
CGTGGTGCACTACACCGGGATGCTTGAAGATGGAAAGAAAGTTGATTCCTCCCGGGACAGAAACAAGCCCTTTAA
GTTTATGCTAGGCAAGCAGGAGGTGATCCGAGGCTGGGAAGAAGGGGTTGCCCAGATGAGTGTGGGTCAGAGA
GCCAAACTGACTATATCTCCAGATTATGCCTATGGTGCCACTGGGCACCCAGGCATCATCCCACCACATGCCACTCT
CGTCTTCGATGTGGAGCTTCTAAAACTGGAATCTGGCGGTGGATCCGGAGTCGACGGATTTGGTGATGTCGGTGC
TCTTGAGAGTTTGAGGGGAAATGCAGATTTGGCTTACATCCTGAGCATGGAGCCCTGTGGCCACTGCCTCATTATC
AACAATGTGAACTTCTGCCGTGAGTCCGGGCTCCGCACCCGCACTGGCTCCAACATCGACTGTGAGAAGTTGCGG

CGTCGCTTCTCCTCGCTGCATTTCATGGTGGAGGTGAAGGGCGACCTGACTGCCAAGAAAATGGTGCTGGCTTTGC
TGGAGCTGGCGCAGCAGGACCACGGTGCTCTGGACTGCTGCGTGGTGGTCATTCTCTCTCACGGCTGTCAGGCCA
GCCACCTGCAGTTCCCAGGGGCTGTCTACGGCACAGATGGATGCCCTGTGTCGGTCGAGAAGATTGTGAACATCT
TCAATGGGACCAGCTGCCCCAGCCTGGGAGGGAAGCCCAAGCTCTTTTTCATCCAGGCCTGTGGTGGGGAGCAGA
AAGACCATGGGTTTGAGGTGGCCTCCACTTCCCCTGAAGACGAGTCCCCTGGCAGTAACCCCGAGCCAGATGCCA
CCCCGTTCCAGGAAGGTTTGAGGACCTTCGACCAGCTGGACGCCATATCTAGTTTGCCCACACCCAGTGACATCTT
TGTGTCCTACTCTACTTTCCCAGGTTTTGTTTCCTGGAGGGACCCCAAGAGTGGCTCCTGGTACGTTGAGACCCTGG
ACGACATCTTTGAGCAGTGGGCTCACTCTGAAGACCTGCAGTCCCTCCTGCTTAGGGTCGCTAATGCTGTTTCGGT
GAAAGGGATTTATAAACAGATGCCTGGTTGCTTTAATTTCCTCCGGAAAAAACTTTTCTTTAAAACATCAGCTAGCA
GAGCCGAGGGCAGGGGAAGTCTTCTAACATGCGGGGACGTGGAGGAAAATCCCGGGCCCATGGGCACCAGCCT
GCTGTGCTGGATGGCCCTGTGCCTGCTGGGCGCCGACCACGCCGATGCCTGCCCCTACAGCAACCCCAGCCTGTG
CAGCGGAGGCGGCGGCAGCGAGCTGCCCACCCAGGGCACCTTCTCCAACGTGTCCACCAACGTGAGCCCAGCCAA
GCCCACCACCACCGCCTGTCCTTATTCCAATCCTTCCCTGTGTAGCGGAGGGGGAGGCAGCCCAGCCCCCAGACCT
CCCACCCCAGCCCCCACCATCGCCAGCCAGCCTCTGAGCCTGAGACCCGAGGCCTGCCGCCCAGCCGCCGGCGGC
GCCGTGCACACCAGAGGCCTGGATTTCGCCTGCGATATCTACATCTGGGCCCCACTGGCCGGCACCTGTGGCGTG
CTGCTGCTGAGCCTGGTGATCACCCTGTACTGCAACCACCGCAACCGCAGGCGCGTGTGCAAGTGCCCCAGGCCC
GTGGTGTGAACGCGTACGTCGCGACCGCGGACATGTACAGAGCTCGAGCGGGATCAATTCCGCCCCCCCCCTAAC
GTTACTGGCCGAAGCCGCTTGGAATAAGGCCGGTGTGCGTTTGTCTATATGTTATTTTCCACCATATTGCCGTCTTT
TGGCAATGTGAGGGCCCGGAAACCTGGCCCTGTCTTCTTGACGAGCATTCCTAGGGGTCTTTCCCCTCTCGCCAAA
GGAATGCAAGGTCTGTTGAATGTCGTGAAGGAAGCAGTTCCTCTGGAAGCTTCTTGAAGACAAACAACGTCTGTA
GCGACCCTTTGCAGGCAGCGGAACCCCCCACCTGGCGACAGGTGCCTCTGCGGCCAAAAGCCACGTGTATAAGAT
ACACCTGCAAAGGCGGCACAACCCCAGTGCCACGTTGTGAGTTGGATAGTTGTGGAAAGAGTCAAATGGCTCTCC
TCAAGCGTATTCAACAAGGGGCTGAAGGATGCCCAGAAGGTACCCCATTGTATGGGATCTGATCTGGGGCCTCGG
TGCACATGCTTTACATGTGTTTAGTCGAGGTTAAAAAACGTCTAGGCCCCCCGAACCACGGGGACGTGGTTTTCCT
TTGAAAAACACGATAATAATATGGTGAGCAAGGGCGAGGAGCTGTTCACCGGGGTGGTGCCCATCCTGGTCGAG
CTGGACGGCGACGTAAACGGCCACAAGTTCAGCGTGTCCGGCGAGGGCGAGGGCGATGCCACCTACGGCAAGCT
GACCCTGAAGTTCATCTGCACCACCGGCAAGCTGCCCGTGCCCTGGCCCACCCTCGTGACCACCCTGACCTACGGC
GTGCAGTGCTTCAGCCGCTACCCCGACCACATGAAGCAGCACGACTTCTTCAAGTCCGCCATGCCCGAAGGCTACG
TCCAGGAGCGCACCATCTTCTTCAAGGACGACGGCAACTACAAGACCCGCGCCGAGGTGAAGTTCGAGGGCGAC
ACCCTGGTGAACCGCATCGAGCTGAAGGGCATCGACTTCAAGGAGGACGGCAACATCCTGGGGCACAAGCTGGA
GTACAACTACAACAGCCACAACGTCTATATCATGGCCGACAAGCAGAAGAACGGCATCAAGGTGAACTTCAAGAT
CCGCCACAACATCGAGGACGGCAGCGTGCAGCTCGCCGACCACTACCAGCAGAACACCCCCATCGGCGACGGCCC
CGTGCTGCTGCCCGACAACCACTACCTGAGCACCCAGTCCGCCCTGAGCAAAGACCCCAACGAGAAGCGCGATCA
CATGGTCCTGCTGGAGTTCGTGACCGCCGCCGGGATCACTCTCGGCATGGACGAGCTGTACAAGTAA

MLEGVQVETISPGDGRTFPKRGQTCVVHYTGMLEDGKKVDSSRDRNKPFKFMLGKQEVIRGWEEGVAQMSVGQRA
KLTISPDYAYGATGHPGIIPPHATLVFDVELLKLESGGGSGVDGFGDVGALESLRGNADLAYILSMEPCGHCLIINNVNFC
RESGLRTRTGSNIDCEKLRRRFSSLHFMVEVKGDLTAKKMVLALLELAQQDHGALDCCVVVILSHGCQASHLQFPGAVY
GTDGCPVSVEKIVNIFNGTSCPSLGGKPKLFFIQACGGEQKDHGFEVASTSPEDESPGSNPEPDATPFQEGLRTFDQLDA
ISSLPTPSDIFVSYSTFPGFVSWRDPKSGSWYVETLDDIFEQWAHSEDLQSLLLRVANAVSVKGIYKQMPGCFNFLRKKL
FFKTSASRAEGRGSLLTCGDVEENPGPMGTSLLCWMALCLLGADHADACPYSNPSLCSGGGGSELPTQGTFSNVSTNV
SPAKPTTTACPYSNPSLCSGGGGSPAPRPPTPAPTIASQPLSLRPEACRPAAGGAVHTRGLDFACDIYIWAPLAGTCGVL
LLSLVITLYCNHRNRRRVCKCPRPVV*
MVSKGEELFTGVVPILVELDGDVNGHKFSVSGEGEGDATYGKLTLKFICTTGKLPVPWPTLVTTLTYGVQCFSRYPDHM
KQHDFFKSAMPEGYVQERTIFFKDDGNYKTRAEVKFEGDTLVNRIELKGIDFKEDGNILGHKLEYNYNSHNVYIMADKQ

KNGIKVNFKIRHNIEDGSVQLADHYQQNTPIGDGPVLLPDNHYLSTQSALSKDPNEKRDHMVLLEFVTAAGITLGMDEL
YK*

>RQR8-I-GFP

ATGGGCACCAGCCTGCTGTGCTGGATGGCCCTGTGCCTGCTGGGCGCCGACCACGCCGATGCCTGCCCCTACAGC
AACCCCAGCCTGTGCAGCGGAGGCGGCGGCAGCGAGCTGCCCACCCAGGGCACCTTCTCCAACGTGTCCACCAAC
GTGAGCCCAGCCAAGCCCACCACCACCGCCTGTCCTTATTCCAATCCTTCCCTGTGTAGCGGAGGGGGAGGCAGCC
CAGCCCCCAGACCTCCCACCCCAGCCCCCACCATCGCCAGCCAGCCTCTGAGCCTGAGACCCGAGGCCTGCCGCCC
AGCCGCCGGCGGCGCCGTGCACACCAGAGGCCTGGATTTCGCCTGCGATATCTACATCTGGGCCCCACTGGCCGG
CACCTGTGGCGTGCTGCTGCTGAGCCTGGTGATCACCCTGTACTGCAACCACCGCAACCGCAGGCGCGTGTGCAA
GTGCCCCAGGCCCGTGGTGTGAACGCGTACGTCGCGACCGCGGACATGTACAGAGCTCGAGCGGGATCAATTCC
GCCCCCCCCCTAACGTTACTGGCCGAAGCCGCTTGGAATAAGGCCGGTGTGCGTTTGTCTATATGTTATTTTCCACC
ATATTGCCGTCTTTTGGCAATGTGAGGGCCCGGAAACCTGGCCCTGTCTTCTTGACGAGCATTCCTAGGGGTCTTT
CCCCTCTCGCCAAAGGAATGCAAGGTCTGTTGAATGTCGTGAAGGAAGCAGTTCCTCTGGAAGCTTCTTGAAGAC
AAACAACGTCTGTAGCGACCCTTTGCAGGCAGCGGAACCCCCCACCTGGCGACAGGTGCCTCTGCGGCCAAAAGC
CACGTGTATAAGATACACCTGCAAAGGCGGCACAACCCCAGTGCCACGTTGTGAGTTGGATAGTTGTGGAAAGAG
TCAAATGGCTCTCCTCAAGCGTATTCAACAAGGGGCTGAAGGATGCCCAGAAGGTACCCCATTGTATGGGATCTG
ATCTGGGGCCTCGGTGCACATGCTTTACATGTGTTTAGTCGAGGTTAAAAAACGTCTAGGCCCCCCGAACCACGGG
GACGTGGTTTTCCTTTGAAAAACACGATAATAATATGGTGAGCAAGGGCGAGGAGCTGTTCACCGGGGTGGTGCC
CATCCTGGTCGAGCTGGACGGCGACGTAAACGGCCACAAGTTCAGCGTGTCCGGCGAGGGCGAGGGCGATGCCA
CCTACGGCAAGCTGACCCTGAAGTTCATCTGCACCACCGGCAAGCTGCCCGTGCCCTGGCCCACCCTCGTGACCAC
CCTGACCTACGGCGTGCAGTGCTTCAGCCGCTACCCCGACCACATGAAGCAGCACGACTTCTTCAAGTCCGCCATG
CCCGAAGGCTACGTCCAGGAGCGCACCATCTTCTTCAAGGACGACGGCAACTACAAGACCCGCGCCGAGGTGAAG
TTCGAGGGCGACACCCTGGTGAACCGCATCGAGCTGAAGGGCATCGACTTCAAGGAGGACGGCAACATCCTGGG
GCACAAGCTGGAGTACAACTACAACAGCCACAACGTCTATATCATGGCCGACAAGCAGAAGAACGGCATCAAGGT
GAACTTCAAGATCCGCCACAACATCGAGGACGGCAGCGTGCAGCTCGCCGACCACTACCAGCAGAACACCCCCAT
CGGCGACGGCCCCGTGCTGCTGCCCGACAACCACTACCTGAGCACCCAGTCCGCCCTGAGCAAAGACCCCAACGA
GAAGCGCGATCACATGGTCCTGCTGGAGTTCGTGACCGCCGCCGGGATCACTCTCGGCATGGACGAGCTGTACAA
GTAA

MGTSLLCWMALCLLGADHADACPYSNPSLCSGGGGSELPTQGTFSNVSTNVSPAKPTTTACPYSNPSLCSGGGGSPAP
RPPTPAPTIASQPLSLRPEACRPAAGGAVHTRGLDFACDIYIWAPLAGTCGVLLLSLVITLYCNHRNRRRVCKCPRPVV*
MVSKGEELFTGVVPILVELDGDVNGHKFSVSGEGEGDATYGKLTLKFICTTGKLPVPWPTLVTTLTYGVQCFSRYPDHM
KQHDFFKSAMPEGYVQERTIFFKDDGNYKTRAEVKFEGDTLVNRIELKGIDFKEDGNILGHKLEYNYNSHNVYIMADKQ
KNGIKVNFKIRHNIEDGSVQLADHYQQNTPIGDGPVLLPDNHYLSTQSALSKDPNEKRDHMVLLEFVTAAGITLGMDEL
YK*

>FKBP-L-FRB-L-ΔCaspase9-T2A-ΔCD19

atggtgggagtgcaggtggaaaccatctccccaggagacgggcgcaccttccccaagcgcggccagacctgcgtggtgcactacaccgggatgcttg
aagatggaaagaaatttgattcctcccgggacagaaacaagccctttaagtttatgctaggcaagcaggaggtgatccgaggctgggaagaaggggg

ttgcccagatgagtgtgggtcagagagccaaactgactatatctccagattatgcctatggtgccactgggcacccaggcatcatcccaccacatgcca
ctctcgtcttcgatgtggagcttctaaaactggaaggggggatctggcggtatcctctggcatgagatgtggcatgaaggcctggaagaggcatctcgtt
tgtactttggggaaaggaacgtgaaaggcatgtttgaggtgctggagcccttgcatgctatgatggaacggggcccccagactctgaaggaaacatcc
tttaatcaggcctatggtcgagatttaatggaggcccaagagtggtgcaggaagtacatgaaatcagggaatgtcaaggacctcacccaagcctggg
acctctattatcatgtgttccgacgaatctcaaagtctggcggtggatccggagtcgacggatttggtgatgtcggtgctcttgagagtttgaggggaaa
tgcagatttggcttacatcctgagcatggagccctgtggccactgcctcattatcaacaatgtgaacttctgccgtgagtccgggctccgcacccgcact
ggctccaacatcgactgtgagaagttgcggcgtcgcttctcctcgctgcatttcatggtggaggtgaagggcgacctgactgccaagaaaatggtgctg
gctttgctggagctggcgcagcaggaccacggtgctctggactgctgcgtggtggtcattctctctcacggctgtcaggccagccacctgcagttcccag
gggctgtctacggcacagatggatgccctgtgtcggtcgagaagattgtgaacatcttcaatgggaccagctgccccagcctgggagggaagcccaa
gctctttttcatccaggcctgtggtggggagcagaaagaccatgggtttgaggtggcctccacttcccctgaagacgagtcccctggcagtaaccccga
gccagatgccaccccgttccaggaaggtttgaggaccttcgaccagctggacgccatatctagtttgcccacacccagtgacatctttgtgtcctactct
actttcccaggttttgtttcctggagggaccccaagagtggctcctggtacgttgagaccctggacgacatctttgagcagtgggctcactctgaagacc
tgcagtccctcctgcttagggtcgctaatgctgtttcggtgaaagggatttataaacagatgcctggttgctttaatttcctccggaaaaaacttttcttta
aaacatcagctagcagagccgagggcaggggaagtcttctaacatgcggggacgtggaggaaaatcccgggcccatgccacctcctcgcctcctctt
cttcctcctcttcctcacccccatggaagtcaggcccgaggaacctctagtggtgaaggtggaagagggagataacgctgtgctgcagtgcctcaagg
ggacctcagatggccccactcagcagctgacctggtctcgggagtccccgcttaaacccttcttaaaactcagcctggggctgccaggcctgggaatcc
acatgaggcccctggccatctggcttttcatcttcaacgtctctcaacagatgggggcttctacctgtgccagccggggcccccctctgagaaggcctg
gcagcctggctggacagtcaatgtggagggcagcggggagctgttccggtggaatgtttcggacctaggtggcctgggctgtggcctgaagaacagg
tcctcagagggccccagctccccttccgggaagctcatgagccccaagctgtatgtgtgggccaaagaccgccctgagatctgggagggagagcctcc
gtgtctcccaccgagggacagcctgaaccagagcctcagccaggacctcaccatggcccctggctccacactctggctgtcctgtggggtaccccctg
actctgtgtccaggggcccccctctcctggacccatgtgcaccccaaggggcctaagtcattgctgagcctagagctgaaggacgatcgcccggccaga
gatatgtgggtaatggagacgggtctgttgttgccccgggccacagctcaagacgctggaaagtattattgtcaccgtggcaacctgaccatgtcattc
cacctggagatcactgctcggccagtactatggcactggctgctgaggactggtggctggaaggtctcagctgtgactttggcttatctgatcttctgcct
gtgttcccttgtgggcattcttcatcttcaaagagccctggtcctgaggaggaaaagaaagcgaatgactgaccccaccaggagattctaa

MVGVQVETISPGDGRTFPKRGQTCVVHYTGMLEDGKKFDSSRDRNKPFKFMLGKQEVIRGWEEGVAQMSVGQRAK
LTISPDYAYGATGHPGIIPPHATLVFDVELLKLEGGSGGILWHEMWHEGLEEASRLYFGERNVKGMFEVLEPLHAMME
RGPQTLKETSFNQAYGRDLMEAQEWCRKYMKSGNVKDLTQAWDLYYHVFRRISKSGGGSGVDGFGDVGALESLRGN
ADLAYILSMEPCGHCLIINNVNFCRESGLRTRTGSNIDCEKLRRRFSSLHFMVEVKGDLTAKKMVLALLELAQQDHGALD
CCVVVILSHGCQASHLQFPGAVYGTDGCPVSVEKIVNIFNGTSCPSLGGKPKLFFIQACGGEQKDHGFEVASTSPEDESP
GSNPEPDATPFQEGLRTFDQLDAISSLPTPSDIFVSYSTFPGFVSWRDPKSGSWYVETLDDIFEQWAHSEDLQSLLLRVA
NAVSVKGIYKQMPGCFNFLRKKLFFKTSASRAEGRGSLLTCGDVEENPGPMPPPRLLFFLLFLTPMEVRPEEPLVVKVEE
GDNAVLQCLKGTSDGPTQQLTWSRESPLKPFLKLSLGLPGLGIHMRPLAIWLFIFNVSQQMGGFYLCQPGPPSEKAWQ
PGWTVNVEGSGELFRWNVSDLGGLGCGLKNRSSEGPSSPSGKLMSPKLYVWAKDRPEIWEGEPPCLPPRDSLNQSLS
QDLTMAPGSTLWLSCGVPPDSVSRGPLSWTHVHPKGPKSLLSLELKDDRPARDMWVMETGLLLPRATAQDAGKYYC
HRGNLTMSFHLEITARPVLWHWLLRTGGWKVSAVTLAYLIFCLCSLVGILHLQRALVLRRKRKRMTDPTRRF*

>F36V-ΔCaspase8-T2A-RQR8

atgctcgaggaaaatcccgggcccggagtgcaggtggaaaccatctccccaggagacgggcgcaccttccccaagcgcggccagacctgcgtggtg
cactacaccgggatgcttgaagatggaaagaaatttgattcctcccgggacagaaacaagccctttaagtttatgctaggcaagcaggaggtgatccg
aggctgggaagaaggggttgcccagatgagtgtgggtcagagagccaaactgactatatctccagattatgcctatggtgccactgggcacccaggc
atcatcccaccacatgccactctcgtcttcgatgtggagcttctaaaactgggggggatctggcggtggctccggcggcggaatcctctggcatgagatg

tggcatgaaggcctggaagaggcatctcgtttgtactttggggaaaggaacgtgaaaggcatgtttgaggtgctggagcccttgcatgctatgatgga
acggggcccccagactctgaaggaaacatcctttaatcaggcctatggtcgagatttaatggaggcccaagagtggtgcaggaagtacatgaaatca
gggaatgtcaaggacctcctccaagcctgggacctctattatcatgtgttccgacgaatctcaaagactagttatccgtacgacgtaccagactacgca
tctggcggtggatccacaatctcggactctccaagagaacaggatagtgaatcacagactttggacaaagtttaccaaatgaaaagcaaacctcggg
gatactgtctgatcatcaacaatcacaattttgcaaaagcacgggagaaagtgcccaaacttcacagcattagggacaggaatggaacacacttgga
tgcaggggctttgaccacgacctttgaagagcttcattttgagatcaagccccacgatgactgcacagtagagcaaatctatgagattttgaaaatcta
ccaactcatggaccacagtaacatggactgcttcatctgctgtatcctctcccatggagacaagggcatcatctatggcactgatggacaggaggcccc
catctatgagctgacatctcagttcactggtttgaagtgcccttcccttgctggaaaacccaaagtgtttttattcaggcttgtcaggggggataactacc
agaaaggtatacctgttgagactgattcagaggagcaaccctatttagaaatggatttatcatcacctcaaacgagatatatcccggatgaggctgac
tttctgctggggatggccactgtgaataactgtgtttcctaccgaaaccctgcagagggaacctggtacatccagtcactttgccagagcctgagagag
cgatgtcctcgaggcgatgatattctcaccatcctgactgaagtgaactatgaagtaagcaacaaggatgacaagaaaaacatggggaaacagatg
cctcagcctactttcacactaagaaaaaaacttgtcttcccttctgatagcgctggaagcggagagggcaggggaagtcttctaacatgcggggacgt
ggaggaaaatcccggcccctctagaatgggcaccagcctgctgtgctggatggccctgtgcctgctgggcgccgaccacgccgatgcctgcccctaca
gcaaccccagcctgtgcagcggaggcggcggcagcgagctgcccacccagggcaccttctccaacgtgtccaccaacgtgagcccagccaagccca
ccaccaccgcctgtccttattccaatccttccctgtgtagcggagggggaggcagcccagcccccagacctcccaccccagcccccaccatcgccagcc
agcctctgagcctgagacccgaggcctgccgcccagccgccggcggcgccgtgcacaccagaggcctggatttcgcctgcgatatctacatctgggcc
ccactggccggcacctgtggcgtgctgctgctgagcctggtgatcaccctgtactgcaaccaccgcaaccgcaggcgcgtgtgcaagtgccccaggcc
cgtggtgtaa

MLEENPGPGVQVETISPGDGRTFPKRGQTCVVHYTGMLEDGKKFDSSRDRNKPFKFMLGKQEVIRGWEEGVAQMSV
GQRAKLTISPDYAYGATGHPGIIPPHATLVFDVELLKLGGSGGGSGGGILWHEMWHEGLEEASRLYFGERNVKGMFEV
LEPLHAMMERGPQTLKETSFNQAYGRDLMEAQEWCRKYMKSGNVKDLLQAWDLYYHVFRRISKTSYPYDVPDYASG
GGSTISDSPREQDSESQTLDKVYQMKSKPRGYCLIINNHNFAKAREKVPKLHSIRDRNGTHLDAGALTTTFEELHFEIKPH
DDCTVEQIYEILKIYQLMDHSNMDCFICCILSHGDKGIIYGTDGQEAPIYELTSQFTGLKCPSLAGKPKVFFIQACQGDNY
QKGIPVETDSEEQPYLEMDLSSPQTRYIPDEADFLLGMATVNNCVSYRNPAEGTWYIQSLCQSLRERCPRGDDILTILTE
VNYEVSNKDDKKNMGKQMPQPTFTLRKKLVFPSDSAGSGEGRGSLLTCGDVEENPGPSRMGTSLLCWMALCLLGAD
HADACPYSNPSLCSGGGGSELPTQGTFSNVSTNVSPAKPTTTACPYSNPSLCSGGGGSPAPRPPTPAPTIASQPLSLRPE
ACRPAAGGAVHTRGLDFACDIYIWAPLAGTCGVLLLSLVITLYCNHRNRRRVCKCPRPVV*
