# Supplementary material for: Combinatorial suicide gene strategies for the safety of cell therapies
Source: Front Immunol. 2022 Sep 14;13:975233. doi: 10.3389/fimmu.2022.975233 (PMC9515659; doi:10.3389/fimmu.2022.975233)
Supplement: Supplementary file 4 [file Table_1.pdf]

Supplemental Table 1: Percentage of killing values for single experiments of Figure 1

NT	ΔiC8-GFP	ΔiC9-ΔCD19	ΔiC8-GFP/ΔiC9-ΔCD19
0.00	74.89	82.36	95.89
0.00	78.51	78.51	94.43
0.00	73.00	76.86	96.20
3.48	22.33	56.27	94.16
0.00	46.71	77.22	92.70
	84.43	65.88	91.95
	81.98	80.25	96.58

NT: non transduced.
