# Supplementary material for: Combinatorial suicide gene strategies for the safety of cell therapies
Source: Front Immunol. 2022 Sep 14;13:975233. doi: 10.3389/fimmu.2022.975233 (PMC9515659; doi:10.3389/fimmu.2022.975233)
Supplement: Supplementary file 5 [file Table_2.pdf]

Supplemental Table 2: Percentage of killing values for single experiments of Figure 3

NT untx	NT BB	NT Siro	NT BB+Siro	ΔiC8-RQR8 untx	ΔiC8-RQR8 BB	ΔiC8-RQR8Siro	ΔiC8-RQR8 BB+Siro	RapaΔiC9 untx	RapaΔiC9 BB	RapaΔiC9 Siro	RapaΔiC9 BB+Siro	ΔiC8-RQR8+Rapa-ΔiC9-ΔCD19 untx	iC8-RQR8+Rapa-iC9 BB	iC8-RQR8+Rapa-iC9 Siro	iC8-RQR8+Rapa-iC9 BB+Siro
				1.00	99.90		99.90					1.00	99.30		99.70
				1.00	99.70		99.10					1.00	99.50		99.90
				1.00	99.10	3.00	99.40					1.00	96.60	99.60	100.00
												1.00	96.50	99.20	99.40
1	22.30	20.90	28.00	1.00	97.60	26.20	98.40	1.00	32.50	92.40	93.20	1.00	99.10	78.70	99.90
1	16.90	25.60	30.50	1.00	98.40	39.50	99.00	1.00	38.30	97.70	95.20	1.00	99.40	83.20	99.90
1	17.80	0.00	7.00	1.00	84.90	1.40	91.40	1.00	20.50	51.40	60.70	1.00	91.90	69.70	96.00

NT: non transduced; untx: untreated
