# Supplementary material for: Combinatorial suicide gene strategies for the safety of cell therapies
Source: Front Immunol. 2022 Sep 14;13:975233. doi: 10.3389/fimmu.2022.975233 (PMC9515659; doi:10.3389/fimmu.2022.975233)
Supplement: Supplementary file 6 [file Table_3.pdf]

Supplemental Table 3: Percentage of killing values for single experiments of Figure 6

	NT			RQR8-I-GFP				iC9-2A-RQR8-I-GFP			
**Untx**											
**BB**	2.00	6.00	1.10	0.00	0.00	0.00	0.00	99.40	99.40	96.30	99.70
**Rituxan**	28.60	1.00	20.80	97.40	73.00	99.00	73.00	91.40	91.40	69.00	
**BB + Rituxan**	0.20	2.40	0.00	99.00	74.80	99.20	74.00	99.20	99.20	96.50	99.90

NT: non transduced, Untx: untreated
